# Standardized diagnostic algorithm for spitzoid lesions aids clinical decision-making and management: a case series from a Swiss reference center

**DOI:** 10.18632/oncotarget.27854

**Published:** 2021-01-19

**Authors:** Marie-Luise Hilbers, Regula Brändli, Beda Mühleisen, Sandra N. Freiberger, Joanna Mangana, Reinhard Dummer

**Affiliations:** ^1^Department of Dermatology, University Hospital Zurich, Zurich, Switzerland; ^2^Department of Dermatopediatrics, Children’s Hospital Zurich, Zurich, Switzerland; ^3^Department of Pathology and Molecular Pathology, University Hospital Zurich, Zurich, Switzerland

**Keywords:** spitzoid nevi, melanocytic lesion, dermatopathology, pediatric dermatology, diagnostic algorithm

## Abstract

Importance: Spitzoid lesions are a group of melanocytic tumors characterized by spindle-like or epithelioid cells with variable malignant potential. While some spitzoid lesions are classified as evidently benign or malignant by clinic and histology, others present with unclear clinical and histological characteristics and are categorized as lesions of intermediate biologic potential. These lesions represent a challenge for pathologists and clinicians alike. No consensus on ancillary diagnostics and clinical management exists. Prediction of their clinical course is difficult. The implementation of ancillary diagnostics is currently subject of extensive discussions.

Observations: We report three cases of spitzoid lesions in three young female patients (3-, 15- and 17 years old) from a single reference center with different clinical and histological manifestations. In each case, uncertain clinical and histological presentation led to the stepwise application of additional diagnostics using immunohistochemistry and a custom next generation sequencing panel optimized for melanocytic lesions (MelArray). Combining ancillary diagnostics helped determine clinical management in all cases by characterizing the biology of these lesions.

Conclusions and Relevance: We illustrate how clinical, histological and molecular features contribute to an optimized management plan in these critical situations and present a possible algorithm for the assessment of spitzoid neoplasms.

## INTRODUCTION

Spitzoid lesions are a diverse group of rapid-growing melanocytic tumors characterized by spindle-like or epithelioid cells. Certain spitzoid lesions show evidently benign or malignant clinic and histology, while others present with unclear characteristics and are therefore categorized as lesions of intermediate biologic potential [1]. These lesions are a challenge for pathologists and clinicians, as there is no consensus on ancillary diagnostics or clinical management [2]. As patients are typically young [2, 3], prediction of the clinical outcome is essential to determine if adjuvant treatment is necessary.

The implementation of ancillary diagnostics such as immunohistochemistry (IHC), comparative genomic hybridization (CGH) and next generation sequencing (NGS) is currently subject of extensive discussions.

The usefulness of IHC in these cases is widely accepted, although there is no consensus on which markers to apply [1, 2, 4]. Garola et al. suggest the use of a panel combining p16, HMB45 and Ki67 stainings [5]. Current research further suggests a stepwise implementation of additional diagnostics including CGH or NGS. However, for all diagnostics, no cut-offs or standardized panel recommendations exist [2].

MelArray is a customized NGS panel optimized for melanocytic lesions that focuses on 190 genes previously reported in melanoma [6].

We use three cases to present the interplay between clinical presentation, pathology and molecular biology and illustrate how this currently influences clinical management.

## CLINICAL CASES

### Patient 1

**Figure 1:**
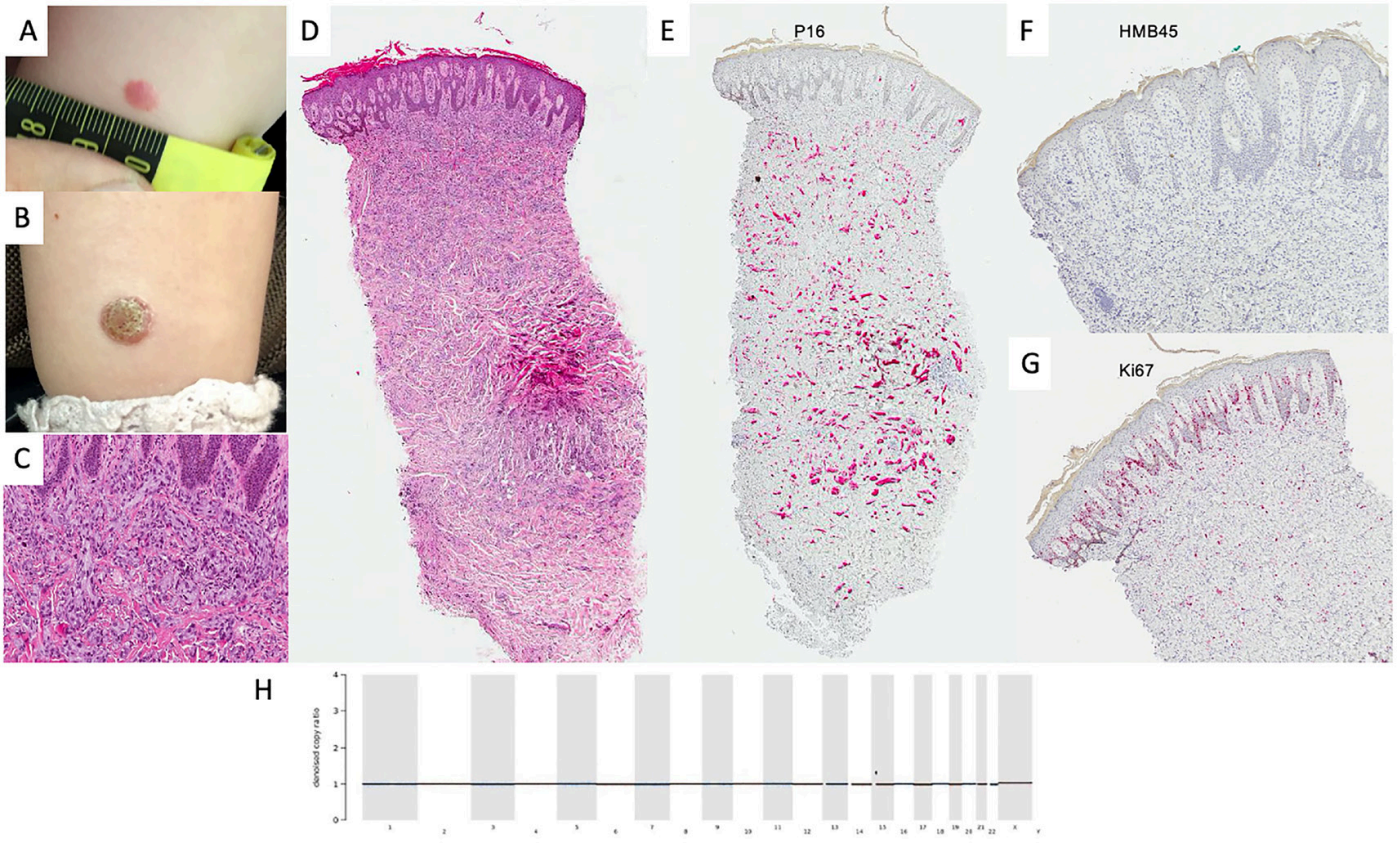
Diagnostics in benign spitzoid lesion. Clinical manifestation of lesion in January 2019 (**A**) and April 2019 (**B**) showing progression from small erythematous macula to verrucous papule. Histologically, the lesion showed pronounced verrucous hyperplasia of the epidermis. In the dermis there is a well demarcated dense infiltration of epitheloid cells with extensive cytoplasm presenting mild mitotic activity (H&E, original magnification 100×) (**C**) and (H&E, original magnification 40×) (**D**). p16 was homogenously positive in melanocytic cells (P16 staining, original magnification 40×) (**E**) and HMB45 was negative in the dermal compartment (H&E, original magnification 40×) (**F**). Ki67 was widely expressed in the dermal-epidermal junction in keratinocytes and some melanocytic cells (Ki67 staining, original magnification 40×) (**G**). Denoised copy ratio depicts the ratio of copy numbers of the tissue analyzed compared to normal tissue gained from the same FFPE block. In this case, no CNVs were seen (**H**). No non-synonymous mutations were detected in the genes included in the MelArray panel.

A 3-year-old female presented with a quickly developing lesion on the right calf. The parents had first noticed a flat, reddish macula at 29 months of age. Within 4 months, it had grown in size and elevation and developed a verrucous aspect. Due to unclear clinical features, the lesion was totally excised in April 2019. Histology, IHC and MelArray were performed (see Figure 1).

Considering atypical clinical presentation and histology with inconspicuous molecular characteristics, clinical follow-ups were recommended after total excision. The patient has been followed up for 9 months without relapse.

### Patient 2

**Figure 2:**
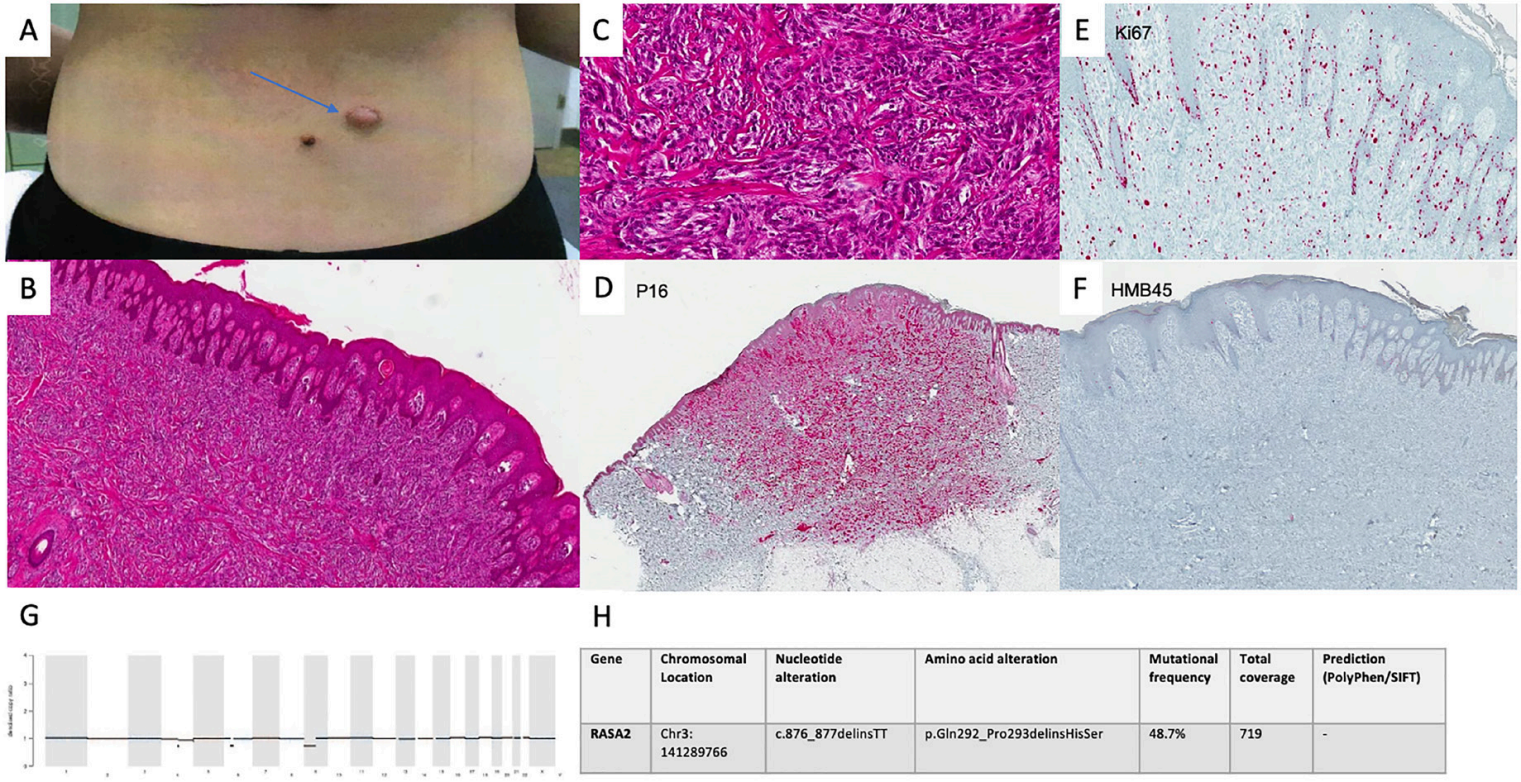
Diagnostics in intermediate spitzoid lesion. Clinical presentation of the lesion in March 2019 (**A**). Conventional histology showed a lesion with nevoid architecture with an acanthotic epithelial reaction pattern. In the dermis there are abundant preferentially spindle cell shaped melanocytic cells presenting disconcerting deep infiltration as well as pleomorphic nuclei and mitotic activity. (H&E, original magnification 40×) (**B**) (H&E, original magnification 100×) (**C**). p16 immunoreactivity was seen as far as the deep dermis (P16 staining, original magnification 40×) (**D**). Ki67 staining confirmed an increased fraction of cells undergoing mitotic proliferation. (Ki67 staining, original magnification 100×) (**E**). HMB45 staining was negative in tumor cells (HMB45 staining, original magnification 40×) (**F**). CNV analysis showed no alterations in MelArray genes (**G**). 7 non-synonymous mutations (11 mut/Mb) were detected in MelArray genes. MelArray also showed a variant in the RASA2 gene (c876_877delinsTT) (**H**).

A 15-year-old female presented with a 13 × 18 mm dome-shaped, pinkish papule on the lower right back. Right inguinal lymphadenopathy was detected concomitantly. The patient reported to have first noticed the skin lesion in October 2017 and an inguinal swelling in February 2019. A biopsy of the skin was taken in March 2019. Histology, IHC and MelArray were performed (see Figure 2).

Based on this diagnostic constellation, a re-excision with 1 cm safety margin was carried out shortly after.

Fine needle aspiration of the enlarged right inguinal lymph node with a diameter of 1 cm and thereafter a modified lymphadenectomy was performed in April 2019.

Histology and IHC of the lymphadenectomy sample showed locoregional spreading.

A PET/CT performed before the operation showed no further manifestations of disease.

The family was informed about the uncertain prognostic outcome. We recommended a close follow-up regimen with clinical assessments every 6 weeks and imaging every 3 months, alternating ultrasound and PET/CT scans and no adjuvant therapy. After 9 months, no relapses have occurred.

### Patient 3

**Figure 3:**
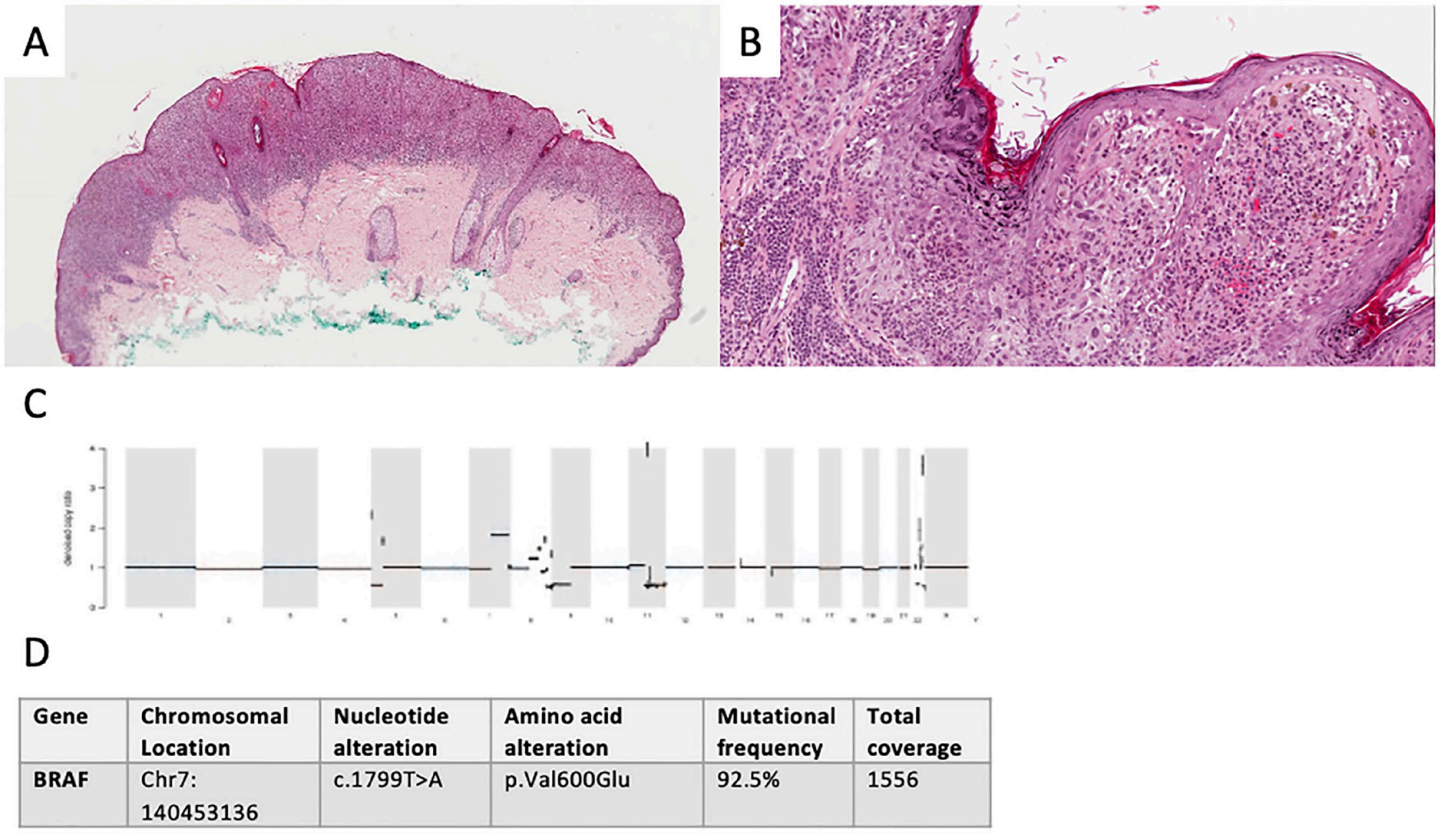
Diagnostics in malignant spitzoid lesion. Clinical presentation of primary not available. Conventional histology showed a polypoid, asymmetrical lesion with irregular shouldering (H&E, original original magnification 40×). (**A**) Melanocytic nests and mutiple single cells, focally lining up in the junction zone present in the atrophic epidermis with slight hyperkeratosis, spindle-like cells with polymorphic nuclei and pagetoid epidermal spread and lack of maturation in the dermis lead to the diagnosis of melanoma with a Breslow depth of 1.9 mm (H&E, original magnification 100×). (**B**) Denoised copy ratio and CNV analysis showed a high number of CNVs (1938 CNVs). (**C**) 11 non-synonymous mutations (17.5 mut/Mb) were seen. A BRAF p.V600E (**D**) mutation as well as alterations in several other melanoma-relevant genes were detected. These included amplifications (such as CCND1 (chromosome 11)) and heterozygous losses (such as loss of CDKN2A (chromosome 9)) (see Supplementary Materials).

A female was referred to our clinic in April 2017 at the age of 14. The girl initially presented with a 15 mm, asymmetrically pigmented lesion on her back. The lesion had been present for years but had increased in size and thickness. Upon biopsy, a distinctly malignant histology and IHC features were seen; MelArray was performed to assess mutational status (see Figure 3, Supplementary Figures 1 and 2).

The clinical management followed current melanoma guidelines: a re-excision with 2 cm safety margin and sentinel lymph node biopsy (SLNB) were performed. The SLNB showed a 2.5 mm metastasis. Adjusted lymphadenectomy was carried out in a peripheral center and was tumor-free. The patient was treated with adjuvant Ipilimumab without complications from August to November 2017. Follow-ups took place every 3 months and showed no recurrence until February 2019, when multiple lymph node, lung and brain metastases were detected during routine diagnostics. The biggest brain metastasis was excised and irradiated stereotactically. Systemic immunotherapy with Ipilimumab and Nivolumab was initiated in March 2019. The patient had to discontinue immunotherapy due to progressive disease and myositis of the ocular muscles after her second and third infusions.

The patient is now undergoing targeted therapy with Dabrafenib and Trametinib since May 2019. She is followed up clinically every month, with imaging every 3 months.

The last PET/CT performed in December 2019 showed complete response of all extracranial lesions and cMRI showed stable disease.

## DISCUSSION AND CONCLUSIONS

Variability of histologic classification [3, 7, 8] and the absence of standardized ancillary diagnostic or therapeutic algorithms [2] create insecurity concerning clinical management and prognosis of patients with spitzoid lesions [1, 9].

In our first patient, unremarkable IHC and molecular patterns lead us to believe that the biology of the lesion was most likely benign and a loose regimen with regular clinical follow-ups was chosen.

The lesion in our second patient showed regular nevus architecture with large cells in conventional histology. Focal Ki-67 activity raised concern. Additionally, NGS showed a variant in the RASA2 gene, a tumor-suppressor for which inactivating mutations are described in 5% of melanomas [10]. To our best knowledge, there are no published reports showing RASA2 alterations in spitzoid lesions, hence the significance of the variant documented in this lesion is unknown.

The frequency of non-synonymous coding mutations suggested an intermediate genetic instability. The constellation of clinical presentation of primary lesion, histology, IHC and NGS, lead us to postulate an intermediate biologic instability of the lesion. Although the tumor had already developed lymph node spreading, we expect a low risk of developing further metastases.

In cases like hers, where prognosis is unpredictable, counseling of patients is crucial. A close follow-up regimen was chosen in agreement with the family, with close clinical follow-ups and frequent imaging. Adjuvant immunotherapy was not recommended.

In our third patient, IHC corroborated with histology, showing distinct signs of malignancy. NGS showed *BRAF* (p.V600E) mutation as well as a variety of other mutations (see Supplementary Materials) and a high amount of copy number variants (CNVs) in the genes analyzed by MelArray. All technologies pointed towards high potential for malignant course of disease, supporting the clinicians’ decision to proceed according to melanoma guidelines including adjuvant immunotherapy. Our primary goal was to define the dignity of these lesions and find a suitable therapeutic strategy, rather than classify them by nomenclature. Nevertheless, we would like to note that, since Spitz nevi only rarely present BRAF (p.V600E) mutations [2, 11], whereas common melanocytic nevi often do [12], we decided to classify this lesion as spitzoid melanoma rather than Spitz melanoma. Hence, we would like to underline that, by using NGS as a part of a standardized diagnostic algorithm, we were able to differentiate between a lesion with traits typical for Spitz tumors versus other types of melanoma by analyzing it’s genetic hallmarks in a clinically and histologically malignant lesion with spitzoid traits.

The combination of specific IHC-staining and NGS gave valuable insight on lesion biology in all cases. As NGS accessibility increases, it is conceivable that it will play a role in routine assessment of biologic potential of spitzoid lesions in the near future. Although the mutational landscape of Spitz tumors differs from cutaneous melanoma [2, 4, 12], many of previously mentioned genetic alterations found in Spitz tumors [2, 11, 13] are mutations, amplifications and losses that are detectable by MelArray (as HRAS mutations, BAP1 mutations, TERTp mutations. see Supplementary Materials). A weakness of the use of this customized NGS panel for characterization of Spitz tumors is the lack of detectability of fusions, which make up a significant part of their genetic landscape [2, 11, 13].

However, information on mutational burden and CNV analysis allows us to estimate the genomic alteration burden that might reflect malignant potential, as multiple mutations and CNVs tend to be more common in lesions with higher malignancy [4, 14, 15].

We are confident that a standardized classification system, which routinely includes IHC and NGS, will give more clarity on spitzoid lesions in the near future [2, 16].

For centers rarely confronted with spitzoid lesions and technologies such as NGS are not readily available, we suggest cases be referred to reference centers for further assessment [2].

**Figure 4:**
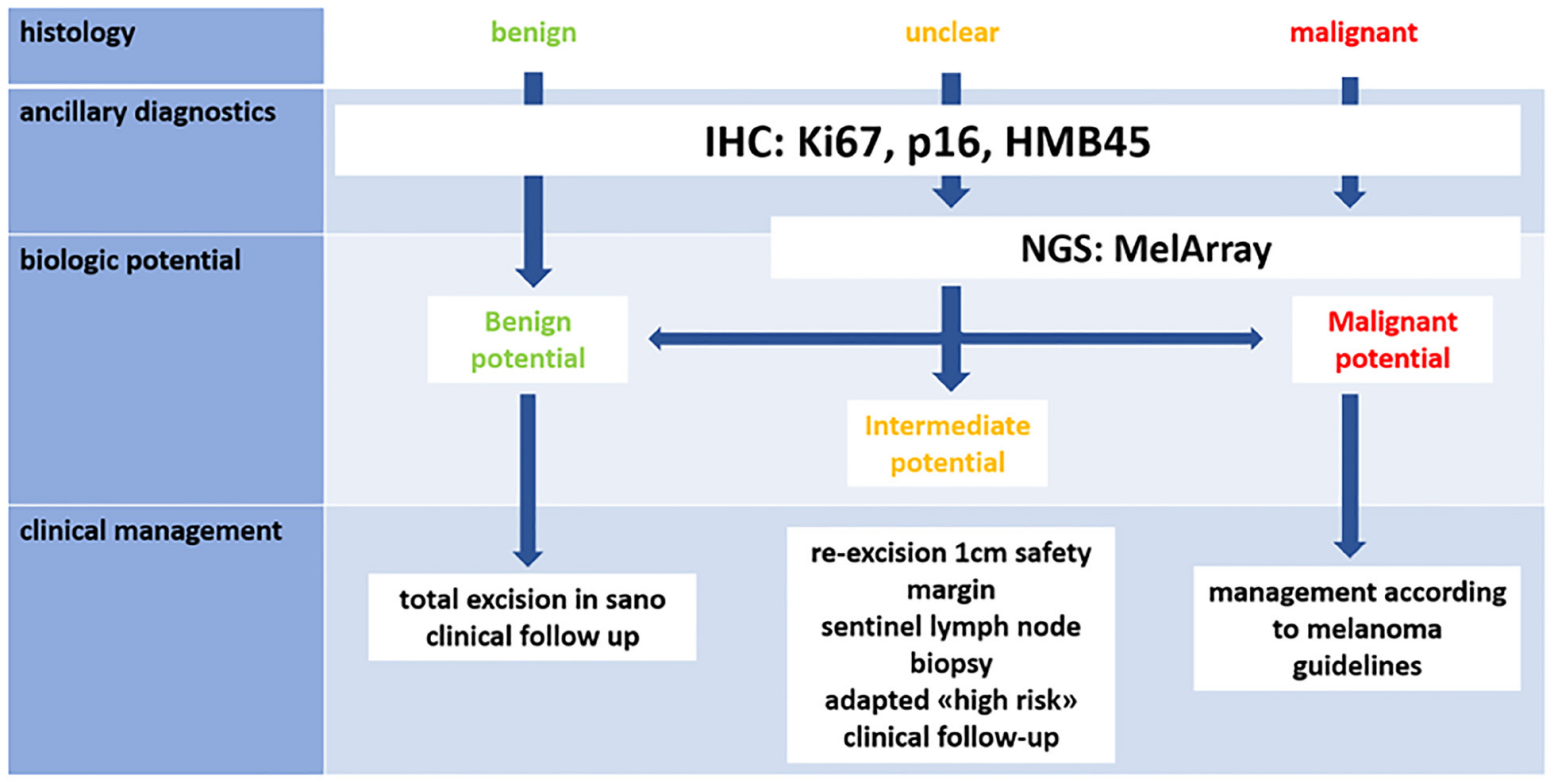
Proposed diagnostic and therapeutic algorithm [2]. IHC combining proliferative and melanocytic markers (for example Ki67, p16, HMB45). If lesion seems benign in histology and IHC, then further analysis might not be needed. If lesion shows unclear or malignant characteristics, MelArray should be performed. Management: probably benign lesions should be totally excised and followed up clinically, intermediate lesions should be excised with a safety margin and potentially receive SLNB, although this is still subject of fierce debate. Malignant lesions should be managed according to melanoma guidelines.

With these cases we portray three different clinical, histological and molecular manifestations of spitzoid lesions. All the information gained impacted the clinical management plan. Here we present a possible algorithm for the classification of biologic potential of spitzoid lesions (Figure 4).

Larger, multi-center validation studies and registries with long-term follow-up are needed to evaluate feasibility and clinical relevance of routine implementation.
