# Addendum: Standardized diagnostic algorithm for spitzoid lesions aids clinical decision-making and management: a case series from a Swiss reference center

**DOI:** 10.18632/oncotarget.28264

**Published:** 2022-08-04

**Authors:** Marie-Luise Hilbers, Regula Brändli, Beda Mühleisen, Sandra N. Freiberger, Joanna Mangana, Reinhard Dummer

**Affiliations:** ^1^Department of Dermatology, University Hospital Zurich, Zurich, Switzerland; ^2^Department of Dermatopediatrics, Children’s Hospital Zurich, Zurich, Switzerland; ^3^Department of Pathology and Molecular Pathology, University Hospital Zurich, Zurich, Switzerland

**Consent was added to this article:** We have informed consent statements from all patients.

Original article: Oncotarget. 2021; 12:125–130. 125-130. https://doi.org/10.18632/oncotarget.27854

