# Supplementary material for: Standardized diagnostic algorithm for spitzoid lesions aids clinical decision-making and management: a case series from a Swiss reference center
Source: Oncotarget. 2021 Jan 19;12(2):125–30. doi: 10.18632/oncotarget.27854 (PMC7825637; doi:10.18632/oncotarget.27854)
Supplement: Supplementary file 1 [file oncotarget-12-125-s001.pdf]

Standardized diagnostic algorithm for spitzoid lesions aids clinical decision-making and management: a case series from a Swiss reference center

SUPPLEMENTARY MATERIALS

Genetic alterations detectable by MelArray. Genetic mutations mentioned in [2, 11, 13] detectable by MelArray: BRAF, HRAS, BAP1, PTEN, TERT promotor, ARID2, CDKN2A, CDK4, CCND1, TP53, AKT1/2/3. Gains and losses of chromosome parts mentioned in [2, 11, 13] detectable by MelArray: 11p gains, 9p21 deletions, 10q23 deletions, 7q34 gains, 3p13 gains, hemizygous losses 6q, 8p, 9p and 10.

**Chr5**	**Chr9**	**Chr11**	**Chr22**
RICTOR	*JAK2*	*TYR*	*PRAME*
SLC45A2	*CDKN2A*	*KMT2A*	*MAPK1*
	CDKN2B	*ATM*	
	TYRP1	*CBL*	

Supplementary Figure 1: Heterozygous loss due to deletions in segments of chromosomes 5, 9, 11 and 22. (Case 3).

**Chr5**	**Chr7**	**Chr11**	**Chr22**
TERT	*AKAP9*	*DPP3*	*PLA2G6*
	CDK6	*CCND1*	*EP300*
	DYNC1I1		
	EZH2		
	POT1		
	MET		
	KMT2C		
	SMO		
	BRAF		
	TRRAP		

Supplementary Figure 2: Low level amplifications due to duplications in segments of chromosomes 5, 7, 11 and 22. (Case 3).
